# Attentional Blink Effects on S-Cone Stimuli

**DOI:** 10.1177/2041669519830103

**Published:** 2019-02-24

**Authors:** Xin Zhang, Feiming Li, Hui Wang, Zhenghuan Mao, HaiFeng Li, Jun Wang, Lei Jia

**Affiliations:** The Children's Hospital, Zhejiang University School of Medicine, Hangzhou, PR China; Department of Psychology, Zhejiang Normal University, Jinhua, PR China; The Children's Hospital, Zhejiang University School of Medicine, Hangzhou, PR China; Department of Psychology, Zhejiang Normal University, Jinhua, PR China

**Keywords:** attentional blink, S-cone and luminance stimuli, koniocellular pathway, masking effects

## Abstract

This study aimed to compare attentional blink (AB) effects on S-cone and on luminance stimuli. Recent research had revealed considerable AB effects not only on high-order visual areas but also on low-order visual areas. Therefore, whether AB formation occurred or not at primary visual cortex must be examined. Previous studies had reported the absence of attention modulation in an early koniocellular pathway driven by S-cone stimuli; therefore, the AB effects on S-cone stimuli would be a strong piece of evidence for late-stage hypothesis at least in the koniocellular pathway. For this study, 12 participants were instructed to identify a centrally presented target (T1) only or to identify either T1 or a peripheral target (T2). The targets were either luminance or S-cone stimuli. As expected, comparable AB effects on S-cone and luminance stimuli were observed. Findings suggested that AB formation through a koniocellular pathway must occur at a later cortical processing stage.

## Introduction

To detect two targets in a rapid serial visual presentation (RSVP) of distractors, the detection ability of the second target (T2) is typically impaired when short stimulus onset asynchrony (SOA) separates two targets. This phenomenon is referred to as attentional blink (AB; Raymond, Shapiro, & Arnell, 1992) and has been reported in many studies (Dux & Marois, 2009; Martens & Wyble, 2010).

The formation of AB is frequently attributed to a slow and capacity-limited attention stage of a two-stage visual processing model (Broadbent & Broadbent, 1987; Chun & Potter, 1995). In this later processing stage, limited attention resources prevent consciously reporting T2 after detecting T1, although both T1 and T2 enter initial unconscious and rapid item categorization stage. Supporting evidence for this theory comes from a behavior fact that T2 can be successfully reported if T1 is ignored; furthermore, electrophysiological (Dellacqua, Doro, Dux, Losier, & Jolicoeur, 2016; Luck, Vogel, & Shapiro, 1996; Maloney, Jayakumar, Levichkina, Pigarev, & Vidyasagar, 2013; Vogel, Luck, & Shapiro, 1998) and functional magnetic resonance imaging findings (Johnston, Linden, & Shapiro, 2012; Marcantoni, Lepage, Beaudoin, Bourgouin, & Richer, 2003; Marois, Yi, & Chun, 2004) suggest that a working memory (WM) load-dependent modulation of neural responses commonly occurs at the fronto-parietal brain regions during AB formation. All these evidence support a WM-based bottleneck and limited attention resources in the later processing stage.

However, considerable evidence reveals that distractors in the RSVP (especially post-T1 distractors) can influence the AB, thereby indicating an alternative to the limited-capacity accounts (Di Lollo, Kawahara, Ghorashi, & Enns, 2005; Nieuwenstein, Chun, Lubbe, & Hooge, 2005; Raymond et al., 1992; Olivers & Watson, 2006). These studies have claimed that an attention gating system, with its suppression ability on distractors, should be highlighted in the AB formation. On the basis of this knowledge, Olivers and Meeter (2008) proposed boost and bounce theory. These authors contended that the attention system in this theory is a rapidly responding gating system (or attentional filter) that seeks to enhance relevant and suppress irrelevant information. Visual items that sufficiently match the target description elicit transient excitatory feedback activity (i.e., a *boost* function), which provides access to WM. However, in the visual stimuli stream of AB, distractors after the target (especially post-T1 distractors) are accidentally boosted, thus resulting in subsequent strong inhibitory feedback response (a *bounce*), which then closes the gate to WM.

Most recent AB research have suggested that AB formation occurs at the late visual processing stage. However, two functional magnetic resonance imaging studies have confirmed that an early visual cortex can be moderated by AB (Stein, Vallines, & Schneider, 2008; Williams, Visser, Cunnington, & Mattingley, 2008). In comparison with prior research, both studies have presented T1 and T2 on different spatial locations and have exploited the retinotopic organization of primary visual cortex. These studies have consistently demonstrated a robust attention modulation in the primary visual cortex, thereby indicating a larger V1 activation for detected T2 than undetected T2. Hein, Alink, Kleinschmidt, and Muller (2009) reported similar reduced brain activities for error-reported T2 in low-order (i.e., V1, V2, and V3 areas) and high-order visual areas (inferior parietal cortex). This significant attention modulation in V1 indicates two possibilities. First, the AB effect on V1 activity may be caused by the feedback from higher level areas. Second, early sensory processing is indicated in the AB formation (Hein et al., 2009; Stein et al., 2008; Williams et al., 2008).

In the present study, we examine the AB effect on S-cone stimuli to investigate these two possibilities in the AB formation. The following benefits of using S-cone stimuli are noted: (a) S-cone stimuli can solely activate anatomically separate koniocellular (KC) pathway. Signals from three visual pathways present in the lateral geniculate nucleus enter V1 in different layers. Most neurons in the magnocellular and parvocellular (MC and PC) pathways target neighboring laminae of layer 4C (4Cα and 4Cβ). However, most KC pathway axons that carry signals initiated in the retinal shortwave-sensitive “S” cones appear to enter a striate cortex in layer 2/3 (Hendry & Reid, 2003; Sincich & Horton, 2005). Signals from KC, MC, and PC pathways are combined no later than two synapses into V1. The investigation of the AB effect on the KC pathway could enrich the current AB literature by determining whether the AB effect is subcortical pathway-independent or not. (b) Differential attentional modulation of cortical responses to S-cone and luminance stimuli. Recent studies have demonstrated the absence of attentional modulation for S-cone but considerable attention modulation for luminance stimuli in V1 (Wang & Wade, 2011). By combining these results, studying the AB effect on S-cone stimuli can differentiate early and late AB stages. If the AB effect is formed at the late processing stage, then comparable AB effects can be observed in S-cone and luminance stimuli. Otherwise, if we observe smaller AB effects on S-cone stimuli than on luminance stimuli, then an early sensory stage in the AB formation can be supported.

## Materials and Methods

### Participants

A total of 12 individuals (1 female, mean age = 21.5 years) participated in this study. All participants had a normal or corrected-to-normal vision (visual acuity of 20/20). The *Smith-Kettlewell Eye Research Institute Review Board* approved this study. All participants provided informed consent before testing. After the experiment, they were thanked and compensated.

### Stimuli and Procedure

**Figure 1.**
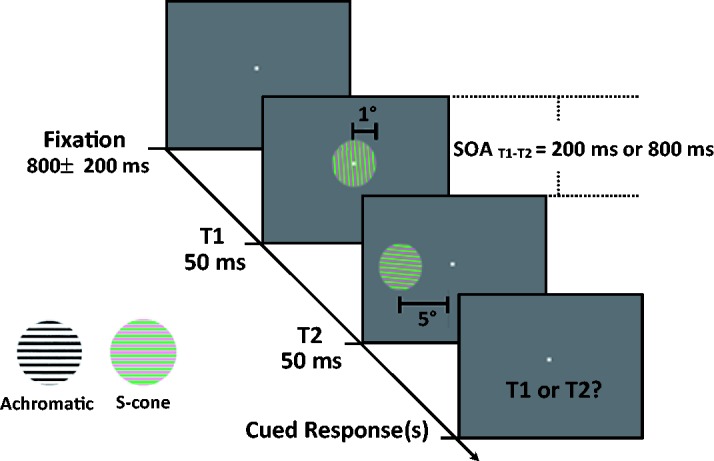
Luminance (i.e., achromatic) and S-cone grating, and an example of the dual-target task trial using S-cone stimuli as T1 and T2. Four stimuli, namely, fixation, T1, T2, and cue of response, were presented in this trial. Horizontal and vertical S-cone gratings tilted clockwise (CW) or counter-clockwise (CCW) off fixations presented as T1 and T2. In the single-target task, only fixation, T2 (acting as T1), and cue of response were presented. In the dual-target task, the participants had to report a T1 or a T2 tilt orientation (50% probability) using the cue at the end of the trial. In the single-target task, they were instructed to ignore T1 and only cued to report the T2 tilt orientation. SOA = stimulus onset asynchrony.

Our paradigm is similar to Hein's et al. (2009) study with certain critical changes, including different spatial locations for T1 and T2. Visual stimuli consisted of two gratings (2° diameter), that is, one aligned with a vertical direction and another aligned with a horizontal direction (Figure 1). The two gratings were presented for 50 ms each and had a fixed presentation order: Vertical one followed by horizontal one. During the experiment, both gratings randomly tilted either clockwise (CW) or counter-clockwise (CCW). The color of the gratings was either achromatic or S-cone. The stimulus was generated by in-house software, running on the Power Macintosh platform. The stimuli were presented using a LaCie Electron Blue II CRT monitor at a frame rate of 100 Hz and a resolution of 800 × 600. The monitor was 70 cm from participants' eyes and had a mean-gray background of 42 cd/m^2^. The procedure for generating cone-isolating stimuli was the same as the one described in a recent study (Wang & Wade, 2011). The participants sat in a dark and quiet room and were instructed to fixate on a central fixation dot of the display.

Each trial started with a gray fixation dot at the center of the screen. This blank screen with a fixation point lasted for a random duration from 600 ms to 1000 ms. Then, the first central presented a grating target (T1). A second grating target (T2) occurred at either 200 ms or 800 ms after the offset of T1 and was randomly presented in either the left or right visual field at an eccentricity of 5°. The participants were required to make a two-alternative forced choice report on the tilt orientations of the gratings (CCW or CW) by pressing “1” (CCW) or “2” (CW) in the keypad. Audio feedback was provided after each button press. Two experimental conditions were followed. In the first condition (*dual target*), the participants were randomly cued to report either a T1 or a T2 tilt orientation at the end of the trial. This activity was conducted to guarantee that the participants concentrate on T1 and T2 and equally attend to their gratings. In the second condition (*single target*), the participants were instructed to ignore T1 and only cued to report the T2 tilt orientation.

An adaptive staircase procedure, namely, “QUEST” (Watson & Pelli, 1983), varied the tilt angle of the gratings to yield 85% correct responses. Each staircase was terminated after 70 trials, and the current estimate of the mean and standard deviation of the tilt angle threshold was recorded. T1 and T2 angle thresholds were recorded for the dual-target condition. Only T2 angle threshold was recorded for the single-target condition. Each subject completed two runs, one using luminance (i.e., achromatic) gratings, whereas the other using S-cone gratings. Each run consisted of four blocks of trials, and the order of trial block presentations was counterbalanced by the number of attended gratings (one or two) and T1 to T2 SOAs (200 ms or 800 ms). Therefore, eight conditions were identified in the psychophysical data.

### AB and Masking Index

To compare behavioral measures directly, we normalized the tilt angle thresholds and compared the magnitude of the AB effect across different types of stimuli (luminance and S-cone).

(1) $$\text{AB}{\text{I}}_{\text{attention}}=\frac{\text{Threshol}{\text{d}}_{\text{T}2\text{\_dual}}-\text{Threshol}{\text{d}}_{\text{T}2\text{\_single}}}{\text{Threshol}{\text{d}}_{\text{T}2\text{\_single}}}$$

This index demonstrated whether a threshold for T2 was modulated by different attention requirements. We calculated this index for long SOA (800 ms) and short SOA (200 ms).

(2) $$\text{AB}{\text{I}}_{\text{SOA}}=\frac{\text{Threshol}{\text{d}}_{\text{T}2\text{\_}800\text{ms}}-\text{Threshol}{\text{d}}_{\text{T}2\text{\_}200\text{ms}}}{\text{Threshol}{\text{d}}_{\text{T}2\text{\_}200\text{ms}}}$$

This index demonstrated whether the threshold for T2 was modulated by different SOAs (800 ms vs. 200 ms).

(3) $$\text{Maskin}{\text{g}}_{\text{backward}}=\frac{\text{Threshol}{\text{d}}_{\text{T}1\text{\_}800\text{ms}}-\text{Threshol}{\text{d}}_{\text{T}1\text{\_}200\text{ms}}}{\text{Threshol}{\text{d}}_{\text{T}1\text{\_}200\text{ms}}}$$

In addition to the two AB indexes (ABI), we also calculated two masking indexes to determine any possible confounding masking effect. In a dual-target condition, these indexes compared T1 thresholds in long SOA (800 ms) and short SOA (200 ms) to determine the possible backward masking effect (T2 mask on T1).

(4) $$\text{Maskin}{\text{g}}_{\text{forward}}=\frac{\text{Threshol}{\text{d}}_{\text{T}2\text{\_}800\text{ms}}-\text{Threshol}{\text{d}}_{\text{T}2\text{\_}200\text{ms}}}{\text{Threshol}{\text{d}}_{\text{T}2\text{\_}200\text{ms}}}$$

In a single-target condition, the index compared T2 thresholds in long SOA (800 ms) and short SOA (200 ms) to determine the possible forward masking effect (T1 mask on T2).

A bootstrapping test with 5,000 replications was used to test (a) whether the indexes were remarkably different from zero (b) and whether amplitudes of ABIs differ between luminance and S-cone gratings. Finally, two ABIs were multiplied by –1 to obtain positive indexes in figures.

## Results

**Figure 2.**
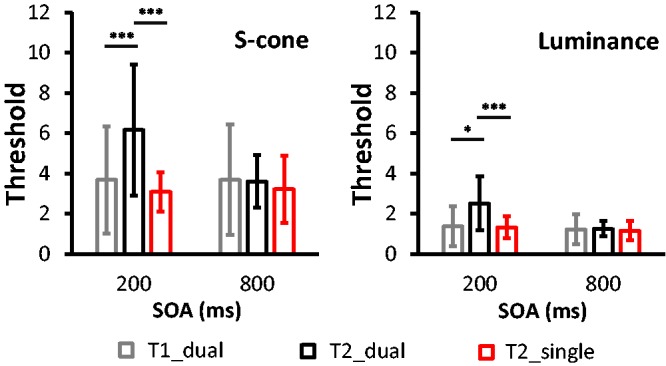
Mean thresholds of T1 and T2 in the single- and dual-target tasks (*p < .05. ***p < .005). SOA = stimulus onset asynchrony.

Figure 2 illustrates the mean thresholds of T1 and T2 of the S-cone and luminance stimuli at different conditions. Using the classical statistical methods in the AB analysis, we examined the thresholds of T1 and T2 in two steps. In the first step, we conducted a 2 (Trial Types: short-SOA trials vs. long-SOA trials) × 2 (T2 in Different Tasks: T2_dual vs. T2_single) nonparametric Friedman two-way analysis of variance (ANOVA) by ranks test on the thresholds of S-cone and luminance stimuli, respectively. As predicted, the ANOVA revealed a significant effect on either S-cone (*p* < .001) stimuli or luminance stimuli (*p* < .001). For the S-cone stimuli, pairwise multiple comparisons using the Wilcoxon signed-rank test revealed a significant difference between T2_dual and T2_single. The difference only appeared at short-SOA trials (*Z* = –3.06, *p* = .002) but disappeared at long-SOA trials (*Z* = –1.02, *p* = .31). In addition, an analysis of the luminance stimuli showed similar effects. The pairwise multiple comparisons revealed a significant difference between T2_dual and T2_single at short-SOA trials (*Z* = –3.06, *p* = .002), but it disappeared at long-SOA trials (*Z* = –.78, *p* = .43).

In the second step, we conducted a 2 (Trial Types: short-SOA trials vs. long-SOA trials) × 2 (T1, T2 in Dual-Target Condition: T1_dual vs. T2_dual) nonparametric Friedman two-way ANOVA by ranks test on the thresholds of S-cone and luminance stimuli, correspondingly. The ANOVA revealed a significant effect on either the S-cone (*p* = .050) stimuli or the luminance stimuli (*p* = .016). For the S-cone stimuli, pairwise multiple comparisons using the Wilcoxon signed-rank test revealed a significant difference between T1_dual and T2_dual. This difference only appeared at short-SOA trials (*Z = *–2.28, *p* = .023), but it disappeared at long-SOA trials (*Z* = –0.16, *p* = .88). Similarly, the pairwise multiple comparisons revealed a significant difference between T1_dual and T2_dual at short-SOA trials (*Z* = –2.43, *p* = .015), but it disappeared at long-SOA trials (*Z* = –0.55, *p* = .58).

**Figure 3.**
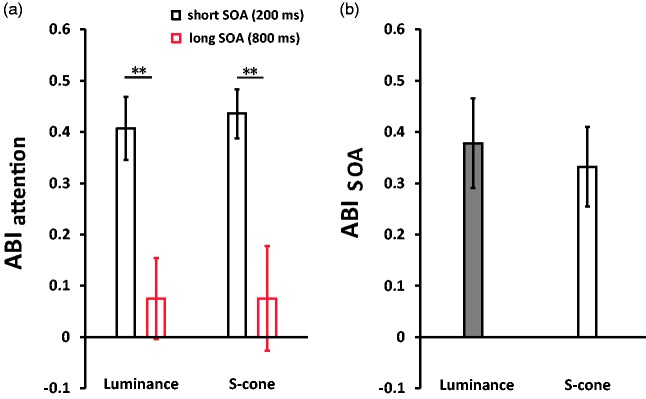
Results of ABIs, namely, ABI_attention_ (a) and ABI_SOA_ (b). **p < .01. SOA = stimulus onset asynchrony; ABI = attentional blink indexes.

These results showed significant AB effects on S-cone and luminance stimuli. Then, we focused on the ABI_attention_ and ABI_SOA_. To examine the potential AB effect elicited by luminance and S-cone stimuli, a 2 (Stimuli Types: luminance vs. S-cone) × 2 (Trial Types: short-SOA trials vs. long-SOA trials) nonparametric Friedman two-way ANOVA by Ranks Test was conducted on ABI_attention_. The ANOVA results revealed a significant effect at *p* = .002. Then, the pairwise multiple comparisons using the Wilcoxon signed-rank test showed significant AB effects (i.e., long-SOA trials vs. short-SOA trials) in luminance (*Z* = –2.76, *p* = .006) and S-cone stimuli (*Z* = –2.67, *p* = .008) trials (Figure 3(a)). However, no effect on the differences between both stimuli types (i.e., luminance vs. S-cone) was found the short-SOA trials or long-SOA trials (*Z*s*<*–.63, *p*s > .05).

**Figure 4.**
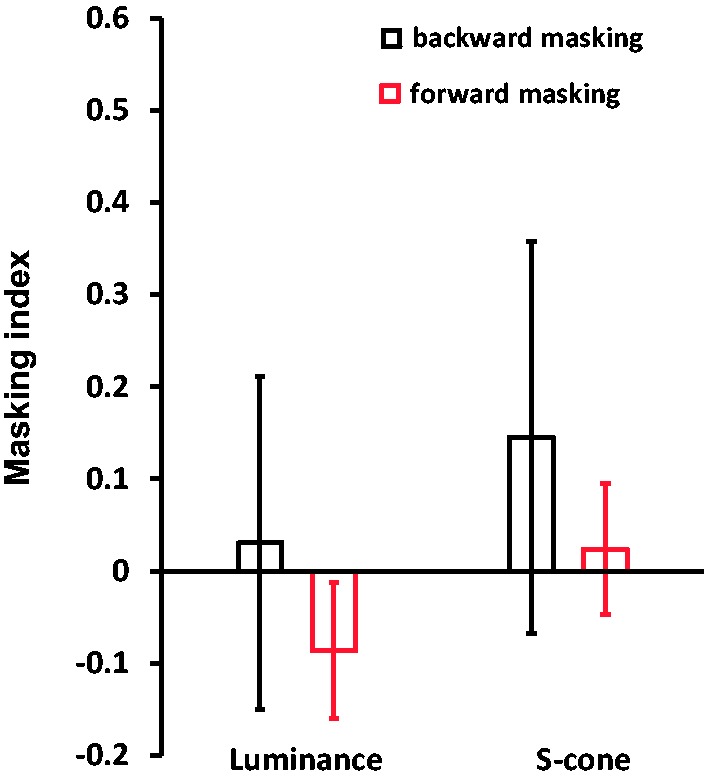
Results of the masking index.

In the next step, the Wilcoxon signed-rank test was conducted on ABI_SOA_ between luminance and S-cone stimuli trials. Figure 3(b) exhibits that no difference was found (*Z* = –.63, *p* > .05).

The probable masking effect between T1 and T2 was then inspected. A 2 (Masking Types: Masking_forward_ vs. Masking_backward_) × 2 (Stimuli Types: luminance vs. S-cone) nonparametric Friedman two-way ANOVA was conducted. This ANOVA did not show any effect (*p* > .05). Furthermore, the Wilcoxon signed-rank test did not reveal any significant effect on backward (T2 on T1) masking, forward (T1 on T2) masking, or amplitudes of the ABIs between luminance and S-cone gratings (*Z*s<–.94, *p*s > .05). Therefore, the AB effects are comparable between luminance and S-cone stimuli.

## Discussion

The present study compared the AB effects on S-cone and luminance stimuli using a modified AB task from a previous study (i.e., Hein et al., 2009) in which no distractors were involved. In comparison with the common RSVP task with distractors, this modified AB task also revealed significant AB effects. The present study found two interesting results. First, luminance and S-cone stimuli demonstrated robust and comparable AB effects given either an attention shift or SOA variance. Second, the absence of significant masking indexes (forward and backward) determined a possible confounding factor that is attributed to the AB effects reported here.

To the best of our knowledge, our study is the first to demonstrate robust AB effects on the KC pathway. The determination of comparable AB effects observed in luminance and S-cone stimuli is important because AB effects can be subcortical visual pathway-independent. Nieuwenhuis, Jepma, La Fors, and Olivers (2008) revealed that AB effects are specific to a magnocellular pathway. In their experiments, variant psychophysical manipulations (e.g., red background) were used to disrupt the magnocellular function. In contrast to their hypothesis, no reduction of AB effects was reported after magnocellular disrupting. Thus, AB effects are indirectly linked to the magnocellular pathway. However, their psychophysical manipulations cannot completely inhibit nor disrupt the magnocellular system. Our study provided a better way to isolate visual pathways; therefore, strengthened their conclusion.

In addition, this study also supports the late processing theory for AB formation. The significant AB effects on S-cone stimuli suggested that attention modulation in early visual areas does not necessarily cause AB effects because the absence of attention modulation has been reported to S-cone stimuli in the primary visual cortex (Wang & Wade, 2011). Similar AB amplitudes to S-cone and luminance implying high-level visual areas play an important role in AB. Therefore, the AB effect on V1 activity should be caused by the feedback from higher level areas.

Furthermore, in this study, forward masking from T1 to T2 does not cause the observed AB effects on luminance and S-cone stimuli for two reasons. First, T1 and T2 were presented in separate spatial locations. Second, when attending to T2 only, no difference was found on thresholds between short and long SOAs (Figure 2). Similar to forward masking, we did not find significant backward masking from T2 to T1. No difference was also observed between short and long SOA in T1 thresholds. The demonstration of robust AB effects on S-cone stimuli extends and contributes to current AB studies. One limitation of the present study is the lack of neuroimaging data to discover neural areas beneath the AB effects on S-cone stimuli. Therefore, we will apply neuroimaging techniques in the future to address this issue.
